# Efficacy of the AS04-adjuvanted HPV-16/18 vaccine in young Chinese women with oncogenic HPV infection at baseline: post-hoc analysis of a randomized controlled trial

**DOI:** 10.1080/21645515.2020.1829411

**Published:** 2020-11-12

**Authors:** Shangying Hu, Xiaoqian Xu, Fengcai Zhu, Ying Hong, Yuemei Hu, Xun Zhang, Qinjing Pan, Wenhua Zhang, Chengfu Zhang, Xiaoping Yang, Jiaxi Yu, Jiahong Zhu, Yejiang Zhu, Feng Chen, Shuang Zhao, Naveen Karkada, Haiwen Tang, Dan Bi, Frank Struyf, Fanghui Zhao

**Affiliations:** a Department of Cancer Epidemiology, National Cancer Center/National Clinical Research Center for Cancer/Cancer Hospital, Chinese Academy of Medical Sciences and Peking Union Medical College, Beijing, China; b Jiangsu Province Center for Disease Prevention and Control, Nanjing, China; c Department of Gynaecology and Obstetrics, Affiliated Drum Tower Hospital of Nanjing University Medical School, Nanjing, China; d Lianshui Center for Disease Prevention and Control, Lianshui, China; e Jintan Center for Disease Prevention and Control, Jintan, China; f Xuzhou Center for Disease Prevention and Control, Xuzhou, China; g Binhai Center for Disease Prevention and Control, Yancheng, China; h GSK, Clinical Research & Development, Wavre, Belgium; i Medical Department, GSK, Shanghai, China; j GSK, Wavre, Belgium at the Time This Analysis Was Performed. Current Affiliation: Janssen Research & Development, Beerse, Belgium

**Keywords:** Human papillomavirus, clinical trial, cervical cancer, China, efficacy, prevention, vaccines, infection

## Abstract

Human papillomavirus (HPV) vaccines are efficacious against HPV infections and associated lesions in women HPV-naïve at vaccination. However, vaccine efficacy (VE) against oncogenic, high-risk HPV (HR-HPV) types in women infected with any other HR-HPV type at first vaccination (baseline) remains unclear. This post-hoc analysis of a phase II/III study (NCT00779766) evaluated AS04-adjuvanted HPV-16/18 (AS04-HPV-16/18) VE against HR-HPV type infection in 871 Chinese women aged 18–25 years over a 72-month follow-up period. Study participants were DNA-negative at baseline to HR-HPV type(s) considered for VE and DNA-positive to any other HR-HPV type. Initial serostatus was not considered. Baseline DNA prevalence was 14.6% for any HR-HPV type and 10.6% excluding HPV-16/18. In the total vaccinated cohort for efficacy, VE against 6-month and 12-month HPV-16/18 persistent infections (PIs) in women DNA-negative to HPV-16/18 but DNA-positive to any other HR-HPV type at baseline was 100.0% (95% Confidence Interval [CI]: 79.8–100.0) and 100.0% (95%CI: 47.2–100.0), respectively. VE against HPV-16/18 incident infections in women DNA-positive to one vaccine type but DNA-negative to the other one at baseline was 66.8% (95%CI: −18.9–92.5). VE against HPV-31/33/45 incident infections, in women DNA-positive to HPV-16/18 and DNA-negative to the considered HPV type at baseline was 71.0% (95%CI: 27.3–89.8). No HPV-16/18 PIs were observed in vaccinated women with non-vaccine HPV A7/A9 species cervical infection at baseline. These findings indicated that women with existing HR-HPV infection at vaccination might still benefit from the AS04-HPV-16/18 vaccine. However, this potential benefit needs further demonstration in the future.

## Introduction

Three prophylactic human papillomavirus (HPV) vaccines have been licensed worldwide for protection against HPV-related diseases and are currently available in over 135 countries. All three vaccines induce strong and sustained protection against anogenital infections and precancerous lesions caused by the vaccine types in subjects HPV-naïve at the time of vaccination.^1–4^ The AS04-adjuvanted HPV-16/18 (AS04-HPV-16/18) vaccine provides some level of protection against oncogenic, high-risk (HR) non-vaccine types HPV-31/33/45 while a limited cross-protection against HPV-31 is observed for the quadrivalent HPV vaccine.^5,6^ Most countries have approved the use of at least one HPV vaccine in women up to age 45 years.^7^ A Chinese domestic HPV vaccine was also approved by the China Food and Drug Administration in December, 2019.^8^

As of June 2020, HPV vaccines were included in 107 countries worldwide as part of national immunization programs (NIP),^9^ targeting mainly adolescent girls. Independently, unvaccinated adult women may seek vaccination services on a user-pay basis. In China, for example, low awareness of HPV infections and related diseases and lack of availability of HPV vaccines in the NIP have led to HPV vaccines being mainly administered to adult women.^10,11^ Adult women are more likely to be sexually active and may thus already have been, or currently be, HPV-infected at the time of first HPV vaccination.

National screening programs for cervical cancer were in place in >144 countries worldwide by 2018.^12^ In some countries where HPV DNA testing is used for cervical cancer screening and prevention efforts, women attending screening service may be aware of an eventual HPV infection before vaccination, although screening for presence of HPV prior to vaccination is not required.^13^ This raised the question about HPV vaccine benefit in this population, especially as the World Health Organization and a growing number of countries are proposing HPV testing as a primary screening tool for cervical cancer.^14^

Until now, most studies focused on subjects HPV-DNA-negative to vaccine types at baseline,^7^ and some of them have demonstrated vaccine efficacy (VE) in women having cleared prior HPV vaccine type infection (seropositive but DNA-negative to vaccine type).^15,16^ It remains unclear whether, among women with cervical HPV infection (HPV DNA-positive) at the time of first vaccination, the residual benefit of preventing infection with other HPV types to which they have not yet been exposed would be sufficient to warrant vaccination. More VE evidence in the HR-HPV infected population is needed for informed decisions by public health authorities and healthcare professionals.

Hence, we performed the current post-hoc analysis to determine VE against infections associated with HPV-16/18 and HPV-31/33/45 types (individually or in combination) in Chinese women aged 18–25 years with cervical HPV infection (DNA-positive) with any other HR-HPV type at the time of first vaccination, irrespective of their serostatus.

**Figure 1.**
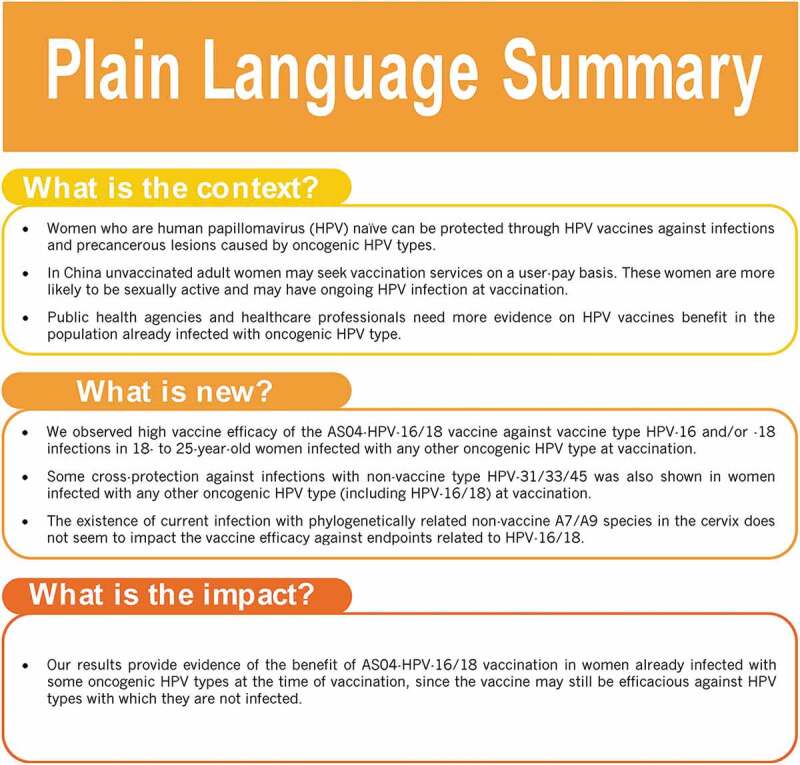
Plain language summary

Figure 1 summarizes the research, clinical relevance and impact of this study on the patient population.

## Methods

### Study design

The primary study (www.ClinicalTrials.gov; NCT00779766) design, methods, and results of event-driven analyses have been previously presented.^17,18^ This post-hoc analysis reports VE against HPV-16/18 and HPV-31/33/45 infections (individually or in combination) in women with cervical infection (DNA-positive) with any HR-HPV type other than the one(s) considered for VE at the time of first vaccination (baseline). Subjects were followed up to 72 months with incident infection and persistent infection (PI) as endpoints.

The trial was carried out in accordance with The Code of Ethics of the World Medical Association (Declaration of Helsinki) and the International Conference on Harmonization Good Clinical Practice guidelines.

### Participants

Healthy women aged 18–25 years were enrolled at four sites (Binhai, Jintan, Lianshui and Xuzhou Centers for Disease Control and Prevention) in Jiangsu Province, China. Women were included in the trial regardless of their HPV DNA status, HPV-16/18 serostatus, or cytology results at baseline. Virgins were not enrolled in the study due to cultural and ethical considerations. Written informed consent was obtained from each participant prior to the performance of any study-specific procedures.

### Procedures

Briefly, study participants were randomized in a 1:1 ratio to receive either the AS04-HPV-16/18 vaccine (*Cervarix*, GSK) or aluminum hydroxide [Al(OH)_3_] at 0, 1, and 6 month intervals in a double-blind manner. AS04 is a proprietary GSK Adjuvant System containing monophosphoryl lipid A (50 μg MPL; produced by GSK) adsorbed on aluminum salt (500 μg Al^3+^). Cervical cytology samples for HPV DNA testing were collected every six months at each study visit. Cervical cytology was tested using the *ThinPrep PapTest* (Cytyc Corporation, Boxborough, MA, USA) and reported according to the Bethesda 2001 classification system. Histopathological analysis was performed by a panel of gynecological pathologists at the Cancer Hospital, Chinese Academy of Medical Sciences (CICAMS), Beijing, China. Final case ascertainment of women assumed to meet the criteria for efficacy endpoints was reviewed in a blinded fashion by an independent review committee. A broad-spectrum PCR assay, SPF10-LiPA25 (version 1 based on licensed *Innogenetics* SPF10 technology; Labo Biomedical Products, Rijswijk, Netherlands) and type-specific PCR for HPV-16 and HPV-18 DNA were used to test cervical samples and biopsy material for HPV DNA from 14 HR-HPV types (16, 18, 31, 33, 35, 39, 45, 51, 52, 56, 58, 59, 66, and 68) and 11 non-HR-HPV types.

### Statistical analysis

Previously published reports of this research disclose the efficacy, immunogenicity, and safety analyses in women DNA-negative at baseline (or both at baseline and Month 6), and seronegative at baseline (or irrespective of serostatus for the considered HPV type) in the according to protocol cohort for efficacy (ATP-E) and the total vaccinated cohort for efficacy (TVC-E).^17,18^ The ATP-E included women with available efficacy data, who received three doses of vaccine or control and had a normal or low-grade cytology at baseline, while TVC-E was similar to ATP-E but included women who received at least one dose of vaccine or control.^17,18^ Here, we specifically report data from women with HR-HPV infection (i.e. HR-HPV DNA-positive, irrespective of HPV serostatus) at baseline in the TVC-E. Subjects were evaluated for subsequent development of HPV infection with the types they were DNA-negative to at baseline.

Based on the analysis of VE against HPV-16/18 and HPV-31/33/45 infection in women DNA-negative to the considered HPV type and DNA-positive to any of the 14 HR-HPV types (i.e. HPV-16/18/31/33/45/39/45/51/52/56/58/59/66/68) at baseline, further analyses were performed in the following three subsets: (i) VE against HPV-16/18 infection in women DNA-negative to the considered HPV type and DNA-positive to any of the other 12 HR-HPV types (i.e. HPV-31/33/45/39/45/51/52/56/58/59/66/68; including HPV-16/18 coinfections) at baseline; (ii) VE against HPV-16/18 and HPV-31/33/45 infection in women DNA-positive to HPV-16/18 and DNA-negative to the considered HPV type at baseline; (iii) VE against HPV-16 infection in women DNA-positive to non-HPV-16 A9 species (i.e. HPV-31/33/35/52/58), and VE against HPV-18 infection in those DNA-positive to non-HPV-18 A7 species (i.e., HPV-39/45/59/68) at baseline. Besides meeting the above criteria for baseline status, women included in the relevant analyses must have samples available after vaccine dose 1 for the evaluation of the virological endpoints.

The evaluated endpoints were incident infection, 6-month PI and 12-month PI associated with specific HPV type(s). Incident infection was defined as the first detection by PCR of an episode of infection by HPV type(s) in a subject previously negative for the considered HPV type(s) and may have been transient or become persistent. Six-month PI was defined as at least two positive HPV DNA PCR assays for the same HPV type(s) with no negative DNA sample between the two positive DNA samples, over an interval of approximately six months. Twelve-month PI was defined as the detection by PCR of the same HPV type(s) at all available timepoints over an interval of approximately 12 months. Due to the limited sample size and the insufficient number of cervical intraepithelial neoplasia of grade 2 or worse (CIN2+) cases, VE against histological endpoints is not presented.

Endpoint cases were calculated as number of subjects reporting at least one event in each group, and VE was calculated using a conditional exact method, as previously described.^17,18^ Case counting started on the day after the first dose and ended at the time of an endpoint event or until the end of the 72-month follow-up. Statistical analyses were carried out with *SAS 9.4* on the SDD platform. A similar method was used for the analysis in the ATP-E cohort.

### Data sharing

Anonymized individual participant data and study documents can be requested for further research from www.clinicalstudydatarequest.com.

## Results

**Figure 2.**
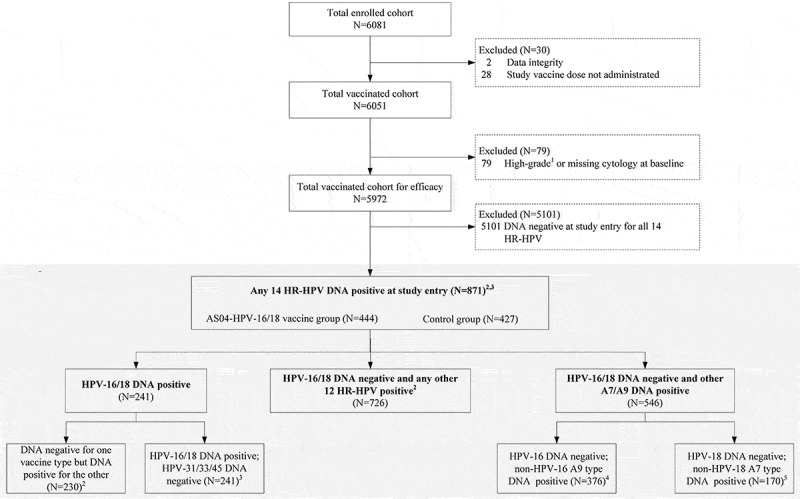
Analysis flowchart

In total, 871 (14.6%) women [444 (14.9%) from the AS04-HPV-16/18 vaccine group and 427 (14.3%) from the control group] from the TVC-E were included in the analysis of ongoing infection with any HR-HPV types, based on the primary objective of this paper (Figure 2). At baseline, 4.0% (241/5,972) of women in the TVC-E were DNA-positive to HPV-16/18 (with or without coinfections with other HR-HPV types), 10.5% (630/5,972) to any other HR-HPV type without HPV-16/18 and coinfections associated with HPV-16 and/or −18, and only 0.2% (11/5,972) were concomitantly positive to both HPV-16 and HPV-18. The mean follow-up time for this cohort was 60.2 months (standard deviation 19.4). Demographic characteristics and HR-HPV distribution in the vaccine and control groups were similar (Table 1).

**Table 1.** Summary of baseline demographic characteristics of women DNA-positive to at least one HR-HPV species at baseline in the TVC-E

	AS04-HPV-16/18 v cohort N = 444	Control cohort N = 427	Total N = 871
Age at first vaccination dose (years, Mean [SD])	23.2 (1.6)	22.8 (1.9)	23.0 (1.8)
Height (cm, mean [SD])	158.8 (5.4)	159.3 (5.3)	159.0 (5.4)
Weight (kg, mean [SD])	54.5 (8.4)	54.5 (8.6)	54.5 (8.5)
BMI (kg/m2, mean [SD])	21.6 (3.1)	21.5 (3.2)	21.5 (3.2)
Race (Chinese heritage, n [%])	473 (100)	451 (100)	924 (100)
Any 14 HR-HPV (n, [%]) (HPV-16/18/31/33/35/39/45/51/52/56/58/59/66/68)	444 (100)	427 (100)	871 (100)
Any 12 HR-HPV^a^ (n, [%]) (HPV-31/33/35/39/45/51/52/56/58/59/66/68)	316 (71.2)	314 (73.5)	630 (72.3)
HPV-16/18/31/33/45(n, [%])	190 (42.8)	166 (38.9)	356 (40.9)
Any non-HPV-16 A9 HR-HPV^b^ (n, [%])	191 (43.0)	185 (43.3)	376 (43.2)
Any non-HPV-18 A7 HR-HPV^c^ (n, [%])	92 (20.7)	78 (18.3)	170 (19.5)
HPV-16 and/or HPV-18 (n, [%])	128 (28.8)	113 (26.5)	241 (27.7)
HPV-16 and HPV-18 (n, [%])	4 (0.9)	7 (1.6)	11 (1.3)
HPV-16 (n, [%])	100 (22.5)	88 (20.6)	188 (21.6)
HPV-18 (n, [%])	32 (7.2)	32 (7.5)	64 (7.3)
HPV-31 (n, [%])	29 (6.5)	25 (5.9)	54 (6.2)
HPV-33 (n, [%])	33 (7.4)	24 (5.6)	57 (6.5)
HPV-45 (n, [%])	19 (4.3)	13 (3.0)	32 (3.7)
HPV-39 (n, [%]) HPV-45 (n, [%]) HPV-51 (n, [%]) HPV-52 (n, [%]) HPV-56 (n, [%]) HPV-58 (n, [%]) HPV-59 (n, [%]) HPV-66 (n, [%]) HPV-68 (n, [%])	46 (10.4) 19 (4.3) 42 (9.5) 109 (24.5) 31 (7.0) 40 (9.0) 6 (1.4) 28 (6.3) 29 (6.5)	33 (7.7) 13 (3.0) 55 (12.9) 123 (28.8) 34 (8.0) 42 (9.8) 16 (3.7) 47 (11.0) 25 (5.9)	79 (9.1) 32 (3.7) 97 (11.1) 232 (26.6) 65 (7.5) 82 (9.4) 22 (2.5) 75 (8.6) 54 (6.2)

^a^Excluding coinfections with HPV-16/18.

^b^Excluding coinfections with HPV-16

^c^Excluding coinfections with HPV-18

AS04-HPV-16/18 v, AS04-adjuvanted HPV-16/18 vaccine; BMI, body mass index; Control, placebo containing Al(OH)_3_; HPV, human papillomavirus; HR-HPV, high-risk human papillomavirus; N, total number of subjects; n (%), number (percentage) of subjects reporting at least one event; SD, standard deviation; TVC-E, total vaccinated cohort for efficacy.

This post-hoc analysis focused on women with existing HR-HPV infection (DNA-positive) at baseline, irrespective of serostatus. For VE against individual HPV type, subjects were DNA-negative to the corresponding HPV type at baseline. For VE against combined types, subjects were DNA-negative to at least one HPV type at baseline (subjects were in the analysis of at least one single type). Besides meeting the above criteria for baseline status, subjects included in the relevant analyses must have sample available after vaccine dose 1 for the evaluation of the virological endpoints, including incident, 6-month and 12-month HPV persistent infections.^1^High-grade cytology included squamous cells cannot exclude high-grade squamous intraepithelial lesions, high-grade squamous intraepithelial lesions, atypical glandular cells, or malignancy^2^VE against incident, 6-month and 12-month persistent infections associated with HPV-16, HPV-18 and HPV-16 and/or HPV-18 was assessed.^3^VE against incident, 6-month and 12-month persistent infections associated with HPV-31, HPV-33, HPV-45, and HPV-31 and/or HPV-33 and/or HPV-45 was assessed.^4^VE against incident, 6-month and 12-month persistent infections only associated with HPV-16 were assessed.^5^VE against incident, 6-month and 12-month persistent infections only associated with HPV-18 were assessed. HR-HPV, high-risk human papillomavirus; N, number of subjects; VE: vaccine efficacy.

### VE against HPV-16/18 infections in women with HR-HPV infections at baseline

Overall, the vaccine had high efficacy against incident infection, 6-month and 12-month PIs associated with HPV-16 and/or HPV-18 in women infected with any of the 14 HR-HPV types at baseline: 74.7% (95% Confidence Interval [CI]: 52.2 to 87.5), 100.0% (95% CI: 79.8 to 100.0) and 100.0% (95% CI: 47.2 to 100.0), respectively. Consistently, high VE was observed in women DNA-negative to HPV-16/18 but DNA-positive to any other 12 HR-HPV types (Table 2).

**Table 2.** Vaccine efficacy against HPV-16/18 infection^a^ stratified by baseline DNA infection status (TVC-E)

VE against		Group	N	n	VE %, (95% CI)	P-value
Women DNA-positive to any of 14 HR-HPV species (HPV-16/18/31/33/35/39/45/51/52/56/58/59/66/68) at Month 0						
**Incident infection with:**	HPV-16/18	AS04-HPV-16/18 v	429	13	74.7 (52.2 to 87.5)	<0.0001
		Control	408	45		
	HPV-16	AS04-HPV-16/18 v	336	8	68.6 (27.8 to 87.8)	0.0034
		Control	329	24		
	HPV-18	AS04-HPV-16/18 v	401	5	81.2 (49.7 to 94.4)	0.0002
		Control	385	24		
**6-month persistent infection with:**	HPV-16/18	AS04-HPV-16/18 v	416	0	100 (79.8 to 100)	<0.0001
		Control	389	18		
	HPV-16	AS04-HPV-16/18 v	327	0	100 (57.9 to 100)	0.0007
		Control	311	10		
	HPV-18	AS04-HPV-16/18 v	388	0	100 (46.5 to 100)	0.0030
		Control	367	8		
**12-month persistent infection with:**	HPV-16/18	AS04-HPV-16/18 v	408	0	100 (47.2 to 100)	0.0028
		Control	378	8		
	HPV-16	AS04-HPV-16/18 v	321	0	100 (−44.2 to 100)	0.0550
		Control	303	4		
	HPV-18	AS04-HPV-16/18 v	381	0	100 (−39.1 to 100)	0.0540
		Control	356	4		
Women DNA-positive to HPV-16/18^b^ at Month 0						
**Incident infection with**^c^:	HPV-16/18	AS04-HPV-16/18 v	121	4	66.8 (−18.9 to 92.5)	0.0921
		Control	102	9		
	HPV-16	AS04-HPV-16/18 v	28	1	61.2 (−646.2 to 99.3)	0.5825
		Control	23	2		
	HPV-18	AS04-HPV-16/18 v	93	3	68.4 (−38.5 to 94.7)	0.1894
		Control	79	7		
Women DNA-positive to any other 12 HR-HPV species (HPV-31/33/35/39/45/51/52/56/58/59/66/68) at Month 0						
**Incident infection with:**	HPV-16/18	AS04-HPV-16/18 v	350	9	79.5 (57.2 to 91.3)	<0.0001
		Control	351	41		
	HPV-16	AS04-HPV-16/18 v	314	7	70.5 (29.0 to 89.3)	0.0043
		Control	317	23		
	HPV-18	AS04-HPV-16/18 v	344	2	91.0 (63.2 to 99.0)	<0.0001
		Control	340	21		
**6-month persistent infection with:**	HPV-16/18	AS04-HPV-16/18 v	338	0	100 (75.4 to 100)	<0.0001
		Control	333	16		
	HPV-16	AS04-HPV-16/18 v	305	0	100 (56.6 to 100)	0.0008
		Control	299	10		
	HPV-18	AS04-HPV-16/18 v	332	0	100 (18.7 to 100)	0.0140
		Control	323	6		
**12-month persistent infection with:**	HPV-16/18	AS04-HPV-16/18 v	332	0	100 (33.1 to 100)	0.0070
		Control	325	7		
	HPV-16	AS04-HPV-16/18 v	300	0	100 (−48.4 to 100)	0.0582
		Control	291	4		
	HPV-18	AS04-HPV-16/18 v	326	0	100 (−133.2 to 100)	0.1181
		Control	315	3		
Women DNA-negative to HPV-16 and DNA-positive to any non-HPV-16 A9 types (HPV-31/33/35/52/58) at Month 0						
**Incident infection with**	HPV-16	AS04-HPV-16/18 v	185	4	69.9 (0.6 to 92.9)	0.0409
		Control	178	12		
**6-month persistent infection with:**		AS04-HPV-16/18 v	179	0	100 (0.8 to 100)	0.0258
		Control	168	5		
**12-month persistent infection with:**		AS04-HPV-16/18 v	176	0	100 (−119.6 to 100)	0.1090
		Control	162	3		
Women DNA-negative to HPV-18 and DNA-positive to any non-HPV-18 A7 types (HPV-39/45/59/68) at Month 0						
**Incident infection with**	HPV-18	AS04-HPV-16/18 v	92	0	100 (45.3 to 100)	0.0037
		Control	78	7		
**6-month persistent infection with:**		AS04-HPV-16/18 v	91	0	100 (−3,137.4 to 100)	0.4518
		Control	75	1		
**12-month persistent infection with:**		AS04-HPV-16/18 v	89	0	-	-
		Control	71	0		

^a^Individual and combined vaccine efficacy against infections with HPV-16 and HPV-18 in women DNA-negative to the considered HPV type at baseline, using conditional exact method. For individual type, subjects were DNA-negative to the corresponding HPV type at Month 0. For combined types, subjects were DNA-negative to at least one HPV type at Month 0 (subjects were in the analysis of at least one single type). Follow-up starts at day after dose 1.

^b^HPV-16/18: subjects DNA-positive to either HPV-16 or HPV-18 at Month 0 and DNA-negative to the other type at Month 0. HPV-16: subjects DNA-positive to HPV-18 at Month 0 and DNA-negative to HPV-16 at Month 0. HPV-18: subjects DNA-positive to HPV-16 at Month 0 and DNA-negative to HPV-18 at Month 0.

**^c^**there were not enough cases to evaluate 6-month or 12-month persistent infection-related endpoints in women DNA-positive to HPV-16/18 at baseline.

AS04-HPV-16/18 v, AS04-adjuvanted HPV-16/18 vaccine; CI, confidence interval; HPV, human papillomavirus; HR-HPV, high-risk human papillomavirus; N, number of subjects included in each group; n, number of subjects reporting at least one event in each group; TVC-E, total vaccinated cohort for efficacy; VE, vaccine efficacy.

We also analyzed the efficacy against infection related to HPV-16/18 in women with HPV-16 or HPV-18 infection at baseline. In 223 women DNA-negative at baseline to one vaccine type but DNA-positive to the other one, VE against incident infection endpoints associated with HPV-16/18 was 66.8% (95% CI: −18.9 to 92.5). There were not enough cases to evaluate 6- or 12-month PI-related endpoints in this subgroup (Table 2).

Two subsets were analyzed to investigate the impact of infection with non-vaccine A9 and A7 HPV species at baseline on VE against virological endpoints associated with HPV-16/18. VE against incident infection, 6- and 12-month PIs associated with HPV-16 in women DNA-positive to non-HPV-16 A9 species at baseline was 69.9% (95% CI: 0.6 to 92.9), 100% (95% CI: 0.8 to 100) and 100% (95% CI: −119.6 to 100), respectively. Similarly, VE against incident and 6-month PI associated with HPV-18 in women DNA-positive to non-HPV-18 A7 species at baseline was 100% (95% CI: 45.3 to 100) and 100% (95% CI: −3,137 to 100), respectively. VE against 12-month HPV-18 PI could not be determined as no cases were reported during the duration of the study (Table 2).

Similar results were obtained for the ATP-E cohort (Supplementary table 1).

### VE against HPV-31/33/45 infections in women with HR-HPV infections at baseline

Cross-protective efficacies against incident infections with HPV-31 and HPV-31/33/45 (any or in combination) in women DNA-positive to any other HR-HPV type at baseline were 63.2% (95% CI: 28.0 to 82.3) and 55.5% (95% CI: 30.3 to 72.1), respectively. VEs against 6-month and 12-month PIs are presented in Table 3.

**Table 3.** Vaccine efficacy against HPV-31/33/45 infection^a^ stratified by baseline DNA infection status (TVC-E)

VE against		Group	N	n	VE %, (95% CI)	P-value
Women DNA-positive to any of 14 HR-HPV species (HPV-16/18/31/33/35/39/45/51/52/56/58/59/66/68) at Month 0						
**Incident infection with:**	HPV-31/33/45	AS04-HPV-16/18 v	433	31	55.5 (30.3 to 72.1)	0.0006
		Control	415	61		
	HPV-31	AS04-HPV-16/18 v	404	13	63.2 (28.0 to 82.3)	0.0032
		Control	391	32		
	HPV-33	AS04-HPV-16/18 v	401	15	47.0 (−3.8 to 73.9)	0.0774
		Control	391	26		
	HPV-45	AS04-HPV-16/18 v	414	8	53.5 (−15.2 to 82.8)	0.0985
		Control	402	16		
**6-month persistent infection with:**	HPV-31/33/45	AS04-HPV-16/18 v	420	19	24.3 (−45.3 to 61.0)	0.4316
		Control	396	23		
	HPV-31	AS04-HPV-16/18 v	393	6	49.8 (−48.0 to 84.8)	0.2234
		Control	374	11		
	HPV-33	AS04-HPV-16/18 v	389	8	−7.8 (−249.1 to 65.9)	1.0000
		Control	373	7		
	HPV-45	AS04-HPV-16/18 v	401	5	22.1 (−206.2 to 81.2)	0.7685
		Control	384	6		
**12-month persistent infection with:**	HPV-31/33/45	AS04-HPV-16/18 v	412	9	25.0 (−99.1 to 72.5)	0.6521
		Control	384	11		
	HPV-31	AS04-HPV-16/18 v	386	1	84.7 (−26.2 to 99.7)	0.0613
		Control	362	6		
	HPV-33	AS04-HPV-16/18 v	382	5	−135.3 (−2,370.4 to 61.5)	0.4522
		Control	361	2		
	HPV-45	AS04-HPV-16/18 v	393	3	6.6 (−597.6 to 87.5)	1.0000
		Control	374	3		
Women DNA-positive to HPV-16/18 at Month 0						
**Incident infection with:**	HPV-31/33/45	AS04-HPV-16/18 v	125	7	71.0 (27.3 to 89.8)	0.0099
		Control	109	18		
	HPV-31	AS04-HPV-16/18 v	121	3	61.4 (−80.5 to 93.8)	0.3104
		Control	106	6		
	HPV-33	AS04-HPV-16/18 v	118	3	70.0 (−24.9 to 94.9)	0.1219
		Control	107	8		
	HPV-45	AS04-HPV-16/18 v	122	2	80.5 (2.2 to 98.0)	0.0488
		Control	108	8		
**6-month persistent infection with:**	HPV-31/33/45	AS04-HPV-16/18 v	121	4	37.3 (−191.4 to 87.6)	0.7376
		Control	107	5		
	HPV-31	AS04-HPV-16/18 v	119	0	100 (−2,921.7 to 100)	0.4664
		Control	104	1		
	HPV-33	AS04-HPV-16/18 v	114	3	−25.4 (−1,401.1 to 85.6)	1.0000
		Control	105	2		
	HPV-45	AS04-HPV-16/18 v	118	1	60.2 (−664.3 to 99.3)	0.6041
		Control	106	2		
**12-month persistent infection with:**	HPV-31/33/45	AS04-HPV-16/18 v	118	4	−59.6 (−1,664 to 77.1)	0.6876
		Control	103	2		
	HPV-31	AS04-HPV-16/18 v	116	0	100 (−2,910.6 to 100)	0.4630
		Control	100	1		
	HPV-33	AS04-HPV-16/18 v	111	3	−150.4 (−13,044.8 to 79.9)	0.6231
		Control	101	1		
	HPV-45	AS04-HPV-16/18 v	115	1	-	-
		Control	102	0		

^a^Individual and combined vaccine efficacy against infections with HPV-31, HPV-33, and HPV-45 in women DNA-negative to the considered HPV type at baseline, using conditional exact method. For individual type, subjects were DNA-negative to the corresponding HPV type at Month 0. For combined types, subjects were DNA-negative to at least one HPV type at Month 0 (subjects were in the analysis of at least one single type). Follow-up starts at day after dose 1.

AS04-HPV-16/18 v, AS04-adjuvanted HPV-16/18 vaccine; CI, confidence interval; HPV, human papillomavirus; HR-HPV, high-risk human papillomavirus; N, number of subjects included in each group; n, number of subjects reporting at least one event in each group; TVC-E, total vaccinated cohort for efficacy; VE, vaccine efficacy

In women with HPV-16/18 infection at baseline, high cross-protective VE against incident infections associated with HPV-31/33/45 was observed: 71.0% (95% CI: 27.3 to 89.8). Cross-protective VE values for 6- and 12-month PIs were 37.3% (95% CI: −191.4 to 87.6) and −59.6% (95% CI: −1,664 to 77.1), respectively (Table 3).

Similar results were obtained for the ATP-E cohort (Supplementary table 2).

### HPV-16/18 associated CIN2± in women with HR-HPV infections at baseline

In all 871 subjects DNA-positive to any 14 HR-HPV types at baseline, irrespective of serostatus, only five CIN2+ cases associated with HPV-16 and/or −18 (all five cases were associated with HPV-16) were reported in subjects DNA-negative to the corresponding HPV type at baseline.

## Discussion

As part of the debate on the added-value of prophylactic HPV vaccination in already sexually active women infected with HPV, this post-hoc analysis supplied data on Chinese 18–25-year-old women who, at the time of first vaccination, had cervical HR-HPV infection (regardless of their serostatus). It evidences that the AS04-HPV-16/18 vaccine may provide preventive benefit to this population.

The FUTURE II (NCT00092534), VIVIANE (NCT00294047) and PATRICIA (NCT00122681) studies have already demonstrated significant VE against vaccine type HPV infection or related neoplasia in subjects with previous or existing HPV infection but DNA-negative for the targeted HPV type at baseline.^16,19,20^ The FUTURE II study, using the quadrivalent HPV vaccine, provided results from women seropositive or DNA-positive to one to three HPV vaccine types before vaccination.^19^ The VIVIANE study, using the AS04-HPV-16/18 vaccine, had a subset analysis consisting of women with a history of previous HPV infection or disease.^20^ However, seropositivity is considered to be associated with prior HPV exposure even though the infection has been cleared, while DNA status informs more about ongoing HR-HPV infection, which significantly affects VE. Moreover, HPV antibody titers are not routinely tested. Thus, our study merely included subjects who were DNA-positive at the time of first vaccination (regardless of their serostatus). The findings provide real-world practical and instructive implications for both clinicians and infected women.

In women DNA-positive to one of the HPV-16/-18 types at baseline but DNA-negative to the other type, our results displayed a statistically non-significant VE against HPV-16/18 incident infections. The PATRICIA study group also analyzed VE against CIN2+ associated with HPV-16/18 in women who were HPV DNA-positive to one vaccine type at baseline but DNA-negative and seronegative to the other vaccine type. The VE was 90.0% (95% CI: 31.8 to 99.8) for HPV-16/18.^16^ Considering DNA status might have more impact on efficacy compared with serostatus, the negative lower bound of the CI for the VE observed in our analysis could be explained by the limited sample size available. It is also possible that efficacy is higher against high-grade lesions than against infections.

As demonstrated in our study, among women DNA-positive to the vaccine types (HPV-16/18), including those simultaneously infected with both types, cross-protection against HPV-31/33/45 incident infections is evident. We also observed decreasing VEs against HPV-31/33/35 infections that became persistence; VE for incident infection > VE for 6-month PI > VE for 12-month PI. These changes in VE estimates may be linked to the underestimation of non-vaccine HPV types in the control group due to the broad-spectrum HPV PCR methodology used. This method is known to potentially underestimate the prevalence of genotypes present at low relative concentrations in multiple infections, a scenario that is more likely to arise in the control group when considering vaccine and cross-protected HPV types.^21^ This bias is stronger for 12-month PI than for 6-month PI and incident infection, and may explain the decreasing VEs observed in this study.^21^

The L1 protein of non-vaccine A9 species (HPV-31/33/35/52/58) and non-vaccine A7 species (HPV-39/45/59/68) display amino acid homology with L1 proteins of HPV-16 and HPV-18 types, respectively.^22^ Based on the phylogenetic relatedness, it is interesting to explore the impact of the preexistence of non-vaccine A7/A9 species infections at first vaccination on VE against endpoints related to HPV-16/18. However, most published studies have focused on cross-protective efficacy and a possible cross-reactive immune response related to A7/A9 species.^22,23^ In contrast, our data indicate high VE against HPV-16 or HPV-18 infections in women DNA-positive to any other non-vaccine HR-HPV A9 or A7 species at vaccination, respectively.

HPV prevalence studies among Chinese women have shown two peaks in HR-HPV infections: a first peak in those aged 15–24 years, soon after the onset of sexual debut, and a second in those aged >40 years.^24^ The second peak is thought to be associated with new HPV exposure and/or the reactivation of a latent infection, and coincides with the peak of cervical cancer incidence observed in women up to 50–55 years old in China.^25^ As evidenced by our study, vaccinating women with preexisting HR-HPV infection could reduce the risk of infection with and transmission of HPV type to which they have not yet been exposed. Moreover, even though HPV vaccines have no therapeutic impact on preexisting infection,^19^ vaccination could prevent eventual re-infection later in life. Vaccination of infected women on an individual basis would confer protection to women from the second attack peak, protect their partners from possible infection and may slow down the progression of the HPV infection^26^ at the scale of the Chinese population. Further evidence is required on this point.

The HPV-FASTER protocol proposed offering HPV vaccination to women in a broad age range (9–45 years) to contribute to faster and greater population impact, based on the results of high VE from phase III clinical trials among adult women. However, vaccination of HR-HPV infected women has been controversial.^27–29^ The results of our analysis lend support to this concept, suggesting vaccinating all women, irrespective of their HPV DNA status. Within the context of global investment and commitments toward the elimination of cervical cancer, this would allow to achieve maximum health benefit and equity to the entire population.

The main strength of our analysis was the specific focus on HR-HPV DNA-positive women at baseline, ignoring confounding factor of serostatus that is moreover not examined routinely, apart from clinical trial testing. An unavoidable limitation is that the findings were generated from a post-hoc analysis of a randomized controlled trial, which was not designed to analyze efficacy in women HR-HPV positive at vaccination, and randomization was not controlled for HPV-infection at baseline. Besides, the small number of women with ongoing HR-HPV infection available for inclusion in the present analysis (i.e., 15% of the total cohort) limits its statistical power. Also, due to insufficient endpoint cases, we evaluated efficacy mainly on a virologic perspective in the form of incident infections, with some subsets based on 6-month and 12-month PIs. However, as demonstrated in previous clinical trials, efficacy against PI is an acceptable surrogate endpoint for efficacy in the prevention of cervical cancer.^30^

In conclusion, our analysis indicated that women with existing HR-HPV infection at the time of first vaccination might still benefit from the AS04-HPV-16/18 vaccine. However, this potential benefit needs further demonstration in the future.

## Supplementary Material

Supplemental Material

## Trademark statement

*Cervarix* is a trademark owned by or licensed to the GSK group of companies.

*ThinPrep PapTest* is a trademark owned by or licensed to Cytyc Corporation.

*Innogenetics* is a trademark owned by or licensed to Innogenetics N.V.

*SAS* is a trademark owned by or licensed to SAS Institute Inc.
