# Supplementary material for: Efficacy of the AS04-adjuvanted HPV-16/18 vaccine in young Chinese women with oncogenic HPV infection at baseline: post-hoc analysis of a randomized controlled trial
Source: Hum Vaccin Immunother. 2020 Nov 12;17(4):955–64. doi: 10.1080/21645515.2020.1829411 (PMC8018349; doi:10.1080/21645515.2020.1829411)
Supplement: Supplemental Material [file KHVI_A_1829411_SM4976.docx]

## **Supplementary Table 1.** Vaccine efficacy against HPV-16/18 infection* stratified by baseline DNA infection status (ATP-E)

| **VE against** | |  | | **Group** | **N** | **n** | **VE %** | **95% CI** | **P-value** |
| --- | --- | --- | --- | --- | --- | --- | --- | --- | --- |
| Women DNA-positive to any of 14 HR-HPV species (HPV-16/18/31/33/35/39/45/51/52/56/58/59/66/68) at Month 0 | | | | | | | | |  |
| **Incident infection with:** | | HPV-16/18 | | AS04-HPV-16/18v | 410 | 10 | 75.6 | (49.8 to 89.2) | <0.0001 |
|  |  |  |  | Control | 381 | 36 |  |  |  |
|  |  | HPV-16 | | AS04-HPV-16/18v | 321 | 7 | 63.6 | (8.7 to 87.2) | 0.0230 |
|  |  |  |  | Control | 302 | 18 |  |  |  |
|  |  | HPV-18 | | AS04-HPV-16/18v | 380 | 3 | 86.5 | (54.5 to 97.4) | 0.0002 |
|  |  |  |  | Control | 358 | 20 |  |  |  |
| **6-month persistent infection with:** | | HPV-16/18 | | AS04-HPV-16/18v | 399 | 0 | 100 | (70.7 to 100) | <0.0001 |
|  |  |  |  | Control | 367 | 13 |  |  |  |
|  |  | HPV-16 | | AS04-HPV-16/18v | 313 | 0 | 100 | (45.4 to 100) | 0.0028 |
|  |  |  |  | Control | 291 | 8 |  |  |  |
|  |  | HPV-18 | | AS04-HPV-16/18v | 370 | 0 | 100 | (0.2 to 100) | 0.0256 |
|  |  |  |  | Control | 344 | 5 |  |  |  |
| **12-month persistent infection with:** | | HPV-16/18 | | AS04-HPV-16/18v | 394 | 0 | 100 | (-36.2 to 100) | 0.0509 |
|  |  |  |  | Control | 358 | 4 |  |  |  |
|  |  | HPV-16 | | AS04-HPV-16/18v | 309 | 0 | 100 | (-398.7 to 100) | 0.2281 |
|  |  |  |  | Control | 283 | 2 |  |  |  |
|  |  | HPV-18 | | AS04-HPV-16/18v | 365 | 0 | 100 | (-387.1 to 100) | 0.2287 |
|  |  |  |  | Control | 335 | 2 |  |  |  |
| Women DNA-positive to HPV-16/18** at Month 0 | | | | | | | | |  |
| **Incident infection with**^†^**:** | | HPV-16/18 | | AS04-HPV-16/18v | 116 | 4 | 50.4 | (-109.3 to 89.7) | 0.3539 |
|  |  |  |  | Control | 96 | 6 |  |  |  |
|  |  | HPV-16 | | AS04-HPV-16/18v | 28 | 1 | 23.3 | (-5,923.2 to 99.0) | 1.0000 |
|  |  |  |  | Control | 21 | 1 |  |  |  |
|  |  | HPV-18 | | AS04-HPV-16/18v | 88 | 3 | 55.8 | (-127.4 to 93.1) | 0.4721 |
|  |  |  |  | Control | 75 | 5 |  |  |  |
| Women DNA-positive to any other 12 HR-HPV species(HPV-31/33/35/39/45/51/52/56/58/59/66/68) at Month 0 | | | | | | | | |  |
| **Incident infection with:** | | HPV-16/18 | | AS04-HPV-16/18v | 333 | 6 | 83.5 | (60.2 to 94.3) | <0.0001 |
|  |  |  |  | Control | 327 | 34 |  |  |  |
|  |  | HPV-16 | | AS04-HPV-16/18v | 299 | 6 | 66.0 | (9.7 to 89.0) | 0.0186 |
|  |  |  |  | Control | 290 | 17 |  |  |  |
|  |  | HPV-18 | | AS04-HPV-16/18v | 325 | 0 | 100 | (79.9 to 100) | <0.0001 |
|  |  |  |  | Control | 316 | 19 |  |  |  |
| **6-month persistent infection with:** | | HPV-16/18 | | AS04-HPV-16/18v | 324 | 0 | 100 | (65.7 to 100) | 0.0002 |
|  |  |  |  | Control | 316 | 12 |  |  |  |
|  |  | HPV-16 | | AS04-HPV-16/18v | 292 | 0 | 100 | (43.8 to 100) | 0.0031 |
|  |  |  |  | Control | 279 | 8 |  |  |  |
|  |  | HPV-18 | | AS04-HPV-16/18v | 316 | 0 | 100 | (-45.6 to 100) | 0.0576 |
|  |  |  |  | Control | 305 | 4 |  |  |  |
| **12-month persistent infection with:** | | HPV-16/18 | | AS04-HPV-16/18v | 319 | 0 | 100 | (-46.0 to 100) | 0.0577 |
|  |  |  |  | Control | 308 | 4 |  |  |  |
|  |  | HPV-16 | | AS04-HPV-16/18v | 288 | 0 | 100 | (-413.5 to 100) | 0.2346 |
|  |  |  |  | Control | 271 | 2 |  |  |  |
|  |  | HPV-18 | | AS04-HPV-16/18v | 311 | 0 | 100 | (-411.9 to 100) | 0.2382 |
|  |  |  |  | Control | 297 | 2 |  |  |  |
| Women DNA-negative to HPV-16 and DNA positive to any non-HPV-16 A9 types (HPV-31/33/35/52/58) at Month 0 | | | | | | | | |  |
| **Incident infection with** | | HPV-16 | | AS04-HPV-16/18v | 177 | 4 | 55.1 | (-67.6 to 90.1) | 0.2441 |
|  |  |  |  | Control | 164 | 8 |  |  |  |
| **6-month persistent infection with:** | |  |  | AS04-HPV-16/18v | 172 | 0 | 100 | (-34.8 to 100) | 0.0495 |
|  |  |  |  | Control | 155 | 4 |  |  |  |
| **12-month persistent infection with:** | |  |  | AS04-HPV-16/18v | 169 | 0 | 100 | (-373.6 to 100) | 0.2203 |
|  |  |  |  | Control | 150 | 2 |  |  |  |
| Women DNA-negative to HPV-18 and DNA positive to any non-HPV-18 A7 types (HPV-39/45/59/68) at Month 0 | | | | | | | | |  |
| **Incident infection with** | | HPV-18 | | AS04-HPV-16/18v | 89 | 0 | 100 | (33.0 to 100) | 0.0074 |
|  |  |  |  | Control | 73 | 6 |  |  |  |
| **6-month persistent infection with:** | |  |  | AS04-HPV-16/18v | 87 | 0 | - | - | - |
|  |  |  |  | Control | 69 | 0 |  |  |  |
| **12-month persistent infection with:** | |  |  | AS04-HPV-16/18v | 86 | 0 | - | - | - |
|  |  | | Control | | 68 | 0 |  |  |  |

*****Individual and combined vaccine efficacy against infections with HPV-16 and HPV-18 in women DNA-negative to the considered HPV type at baseline, using conditional exact method. For individual type, subjects were DNA-negative to the corresponding HPV type at Month 0 and Month 6. For combined types, the subjects were DNA-negative to at least one HPV type at Month 0 and Month 6 (subjects were in the analysis of at least one single type). Follow-up starts at day after dose 3.

**HPV-16/18: subjects DNA-positive to either HPV-16 or HPV-18 at Month 0 and DNA-negative to the other type at Month 0 and Month 6. HPV-16: subjects DNA-positive to HPV-18 at Month 0 and DNA-negative to HPV-16 at Month 0 and Month 6. HPV-18: subjects DNA-positive to HPV-16 at Month 0 and DNA-negative to HPV-18 at Month 0 and Month 6.

^†^There were not enough cases to evaluate 6-month or 12-month persistent infection related endpoints in women DNA-positive to HPV-16/18.

AS04-HPV-16/18v, AS04-adjuvanted HPV-16/18 vaccine; ATP-E, according to protocol efficacy cohort; CI, confidence interval; HPV, human papillomavirus; HR-HPV, high-risk human papillomavirus; N, number of subjects included in each group; n, number of subjects reporting at least one event in each group; VE, vaccine efficacy.

## **Supplementary Table 2.** Vaccine efficacy against HPV-31/33/45 infection* stratified by baseline DNA infection status (ATP-E)

| **VE against** |  | **Group** | **N** | **n** | **VE %** | **95% CI** | **p-Value** | |
| --- | --- | --- | --- | --- | --- | --- | --- | --- |
| Women DNA-positive to any of 14 HR-HPV species (HPV-16/18/31/33/35/39/45/51/52/56/58/59/66/68) at Month 0 | | | | | | | |  |
| **Incident infection with:** | HPV-31/33/45 | AS04-HPV-16/18v | 414 | 23 | 52.5 | (19.5 to 72.7) | 0.0046 | |
|  |  | Control | 388 | 43 |  |  |  | |
|  | HPV-31 | AS04-HPV-16/18v | 383 | 9 | 66.5 | (25.2 to 86.3) | 0.0043 | |
|  |  | Control | 359 | 24 |  |  |  | |
|  | HPV-33 | AS04-HPV-16/18v | 381 | 12 | 45.7 | (-16.7 to 75.8) | 0.1471 | |
|  |  | Control | 360 | 20 |  |  |  | |
|  | HPV-45 | AS04-HPV-16/18v | 393 | 6 | 30.6 | (-128.1 to 80.2) | 0.5950 | |
|  |  | Control | 370 | 8 |  |  |  | |
| **6-month persistent infection with:** | HPV-31/33/45 | AS04-HPV-16/18v | 403 | 11 | 28.4 | (-69.7 to 70.6) | 0.5425 | |
|  |  | Control | 373 | 14 |  |  |  | |
|  | HPV-31 | AS04-HPV-16/18v | 374 | 3 | 69.9 | (-20.5 to 94.8) | 0.0800 | |
|  |  | Control | 346 | 9 |  |  |  | |
|  | HPV-33 | AS04-HPV-16/18v | 371 | 5 | 7.8 | (-300.8 to 78.8) | 7.8 | |
|  |  | Control | 345 | 5 |  |  |  | |
|  | HPV-45 | AS04-HPV-16/18v | 382 | 3 | -177.9 | (-14,489.0 to 77.7) | 0.6248 | |
|  |  | Control | 358 | 1 |  |  |  | |
| **12-month persistent infection with:** | HPV-31/33/45 | AS04-HPV-16/18v | 397 | 6 | 8.9 | (-240.8 to 75.6) | 1.0000 | |
|  |  | Control | 363 | 6 |  |  |  | |
|  | HPV-31 | AS04-HPV-16/18v | 369 | 1 | 81.9 | (-61.4 to 99.6) | 0.1086 | |
|  |  | Control | 337 | 5 |  |  |  | |
|  | HPV-33 | AS04-HPV-16/18v | 367 | 3 | -175.9 | (-14,383.8 to 77.9) | 0.6253 | |
|  |  | Control | 337 | 1 |  |  |  | |
|  | HPV-45 | AS04-HPV-16/18v | 376 | 2 | - | (- to 82.7) | 0.5001 | |
|  |  | Control | 348 | 0 |  |  |  | |
| Women DNA-positive to HPV-16/18 at Month 0 | | | | | | | |  |
| **Incident infection with:** | HPV-31/33/45 | AS04-HPV-16/18v | 120 | 6 | 58.5 | (-22.3 to 87.4) | 0.1344 | |
|  |  | Control | 104 | 11 |  |  |  | |
|  | HPV-31 | AS04-HPV-16/18v | 118 | 3 | 24.7 | (-462.1 to 89.9) | 1.0000 | |
|  |  | Control | 98 | 3 |  |  |  | |
|  | HPV-33 | AS04-HPV-16/18v | 114 | 3 | 61.3 | (-81.0 to 93.7) | 0.3100 | |
|  |  | Control | 100 | 6 |  |  |  | |
|  | HPV-45 | AS04-HPV-16/18v | 116 | 1 | 74.3 | (-219.8 to 99.5) | 0.3364 | |
|  |  | Control | 99 | 3 |  |  |  | |
| **6-month persistent infection with:** | HPV-31/33/45 | AS04-HPV-16/18v | 116 | 3 | -136.3 | (-12,304.5 to 81.0) | 0.6257 | |
|  |  | Control | 100 | 1 |  |  |  | |
|  | HPV-31 | AS04-HPV-16/18v | 114 | 0 | - | - | - | |
|  |  | Control | 95 | 0 |  |  |  | |
|  | HPV-33 | AS04-HPV-16/18v | 110 | 3 | -140.1 | (-12,502.3 to 80.7) | 0.6248 | |
|  |  | Control | 96 | 1 |  |  |  | |
|  | HPV-45 | AS04-HPV-16/18v | 112 | 0 | - | - | - | |
|  |  | Control | 96 | 0 |  |  |  | |
| **12-month persistent infection with:** | HPV-31/33/45 | AS04-HPV-16/18v | 114 | 3 | - | (- to 67.3) | 0.2507 | |
|  |  | Control | 98 | 0 |  |  |  | |
|  | HPV-31 | AS04-HPV-16/18v | 113 | 0 | - | - | - | |
|  |  | Control | 93 | 0 |  |  |  | |
|  | HPV-33 | AS04-HPV-16/18v | 109 | 3 | - | (- to 66.9) | 0.2504 | |
|  |  | Control | 94 | 0 |  |  |  | |
|  | HPV-45 | AS04-HPV-16/18v | 110 | 0 | - | - | - | |
|  |  | Control | 94 | 0 |  |  |  | |

*Individual and combined vaccine efficacy against infections with HPV-31, HPV-33, and HPV-45 in women DNA-negative to the considered HPV type at baseline, using conditional exact method. For individual type, subjects were DNA-negative to the corresponding HPV type at Month 0 and Month 6. For combined types, the subjects were DNA-negative to at least one HPV type at Month 0 and Month 6 (subjects were in the analysis of at least one single type). Follow-up starts at day after dose 3.

AS04-HPV-16/18v, AS04-adjuvanted HPV-16/18 vaccine; ATP-E, according to protocol efficacy cohort; CI, confidence interval; HPV, human papillomavirus; HR-HPV, high-risk human papillomavirus; N, number of subjects included in each group; n, number of subjects reporting at least one event in each group; VE, vaccine efficacy.
